# Intracranial Pressure During Mobilization in a Patient With Postoperative Putaminal Hemorrhage: A Case Report

**DOI:** 10.7759/cureus.80783

**Published:** 2025-03-18

**Authors:** Hikaru Takara

**Affiliations:** 1 Rehabiltation, Naha City Hospital, Okinawa, JPN

**Keywords:** intracranial pressure, mobilization, neuro-critical care, physical therapy, putaminal hemorrhage, rehabilitation

## Abstract

Rehabilitation and mobilization for patients with severe stroke, including postsurgical cases, are considered feasible if the intracranial pressure (ICP) remains below 20 mmHg. However, to the best of our knowledge, no study has evaluated real-time ICP changes during mobilization in patients with postoperative putaminal hemorrhage. In this case report, we detail the ICP fluctuations observed during mobilization in a patient with postoperative putaminal hemorrhage. A 47-year-old man was diagnosed with left putaminal hemorrhage and underwent emergency craniotomy for hematoma evacuation (day 1). Postoperatively, the patient was managed using continuous ICP monitoring. Mobilization (sitting on the edge of the bed) began on day 3, during which time the ICP was evaluated. The preintervention ICP was well controlled, ranging from 13 to 15 mmHg. During the transition to the sitting position, the patient attempted to rise on his own, leading to a transient ICP elevation exceeding 20 mmHg for approximately 20 seconds. While maintaining the sitting position, the ICP stabilized at approximately 5 mmHg without sustained elevation. The blood pressure, heart rate, and other vital signs remained stable throughout the intervention period. Our findings suggest that mobilization to a sitting position may be feasible without inducing sustained ICP elevation when ICP is well controlled.

## Introduction

Early rehabilitation and mobilization in patients with stroke are recommended to improve functional outcomes and prevent complications such as pneumonia [1,2]. However, in severe cases and postsurgical patients, intracranial pressure (ICP) is a critical concern and may limit rehabilitation interventions [3]. In patients requiring ICP monitoring, careful assessment of ICP changes during rehabilitation is essential [4]. Specifically, mobilization involving postural changes may trigger ICP fluctuations that require close attention.

Mobilization is considered feasible when the ICP is controlled below 20 mmHg [5]. Previous studies on the mobilization for subarachnoid and intracerebral hemorrhages have reported no significant elevation in ICP [6,7]. However, these studies evaluated ICP before and after mobilization without reporting real-time changes during the mobilization. To the best of our knowledge, no study has evaluated real-time changes in ICP during mobilization. Understanding the characteristics of ICP fluctuations during mobilization (e.g., limb movements in a sitting position, sitting-up motion, and standing-up motion) may provide insights into which movements are safe and which require caution. Assessing ICP during mobilization is as important as preintervention and postintervention ICP evaluations because understanding these fluctuations could strengthen evidence for the safety of early mobilization.

Herein, we examined real-time ICP changes during mobilization in a patient with putaminal hemorrhage managed by ICP monitoring.

## Case presentation

**Figure 1:**
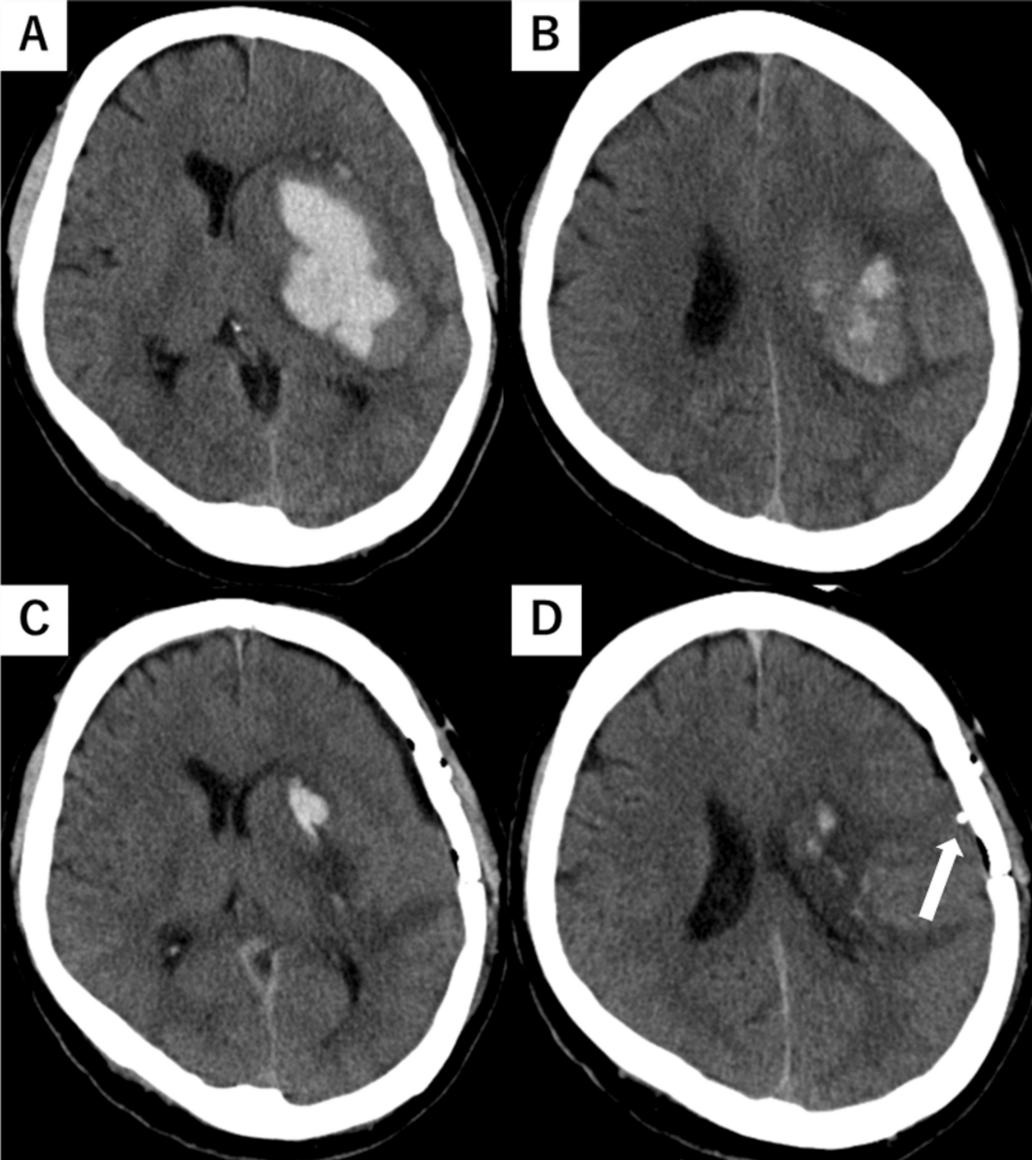
Preoperative and postoperative brain CT. (A–B) Preoperative brain CT. The hematoma is located in the putamen, and measures approximately 60 mL and is associated with a mild midline shift and obscuration of the left sulcus. The hematoma was measured as 6.7 cm (length) × 5.5 cm (width) × 3.3 cm (height). (C–D) Postoperative brain CT. The hematoma in the putamen has been removed, while a hematoma remains in the left caudate nucleus. The midline shift has improved; however, the obscuration of the left sulcus persists. ICP devices(probe) are indicated by white arrows. CT, computed tomography.

**Figure 2:**
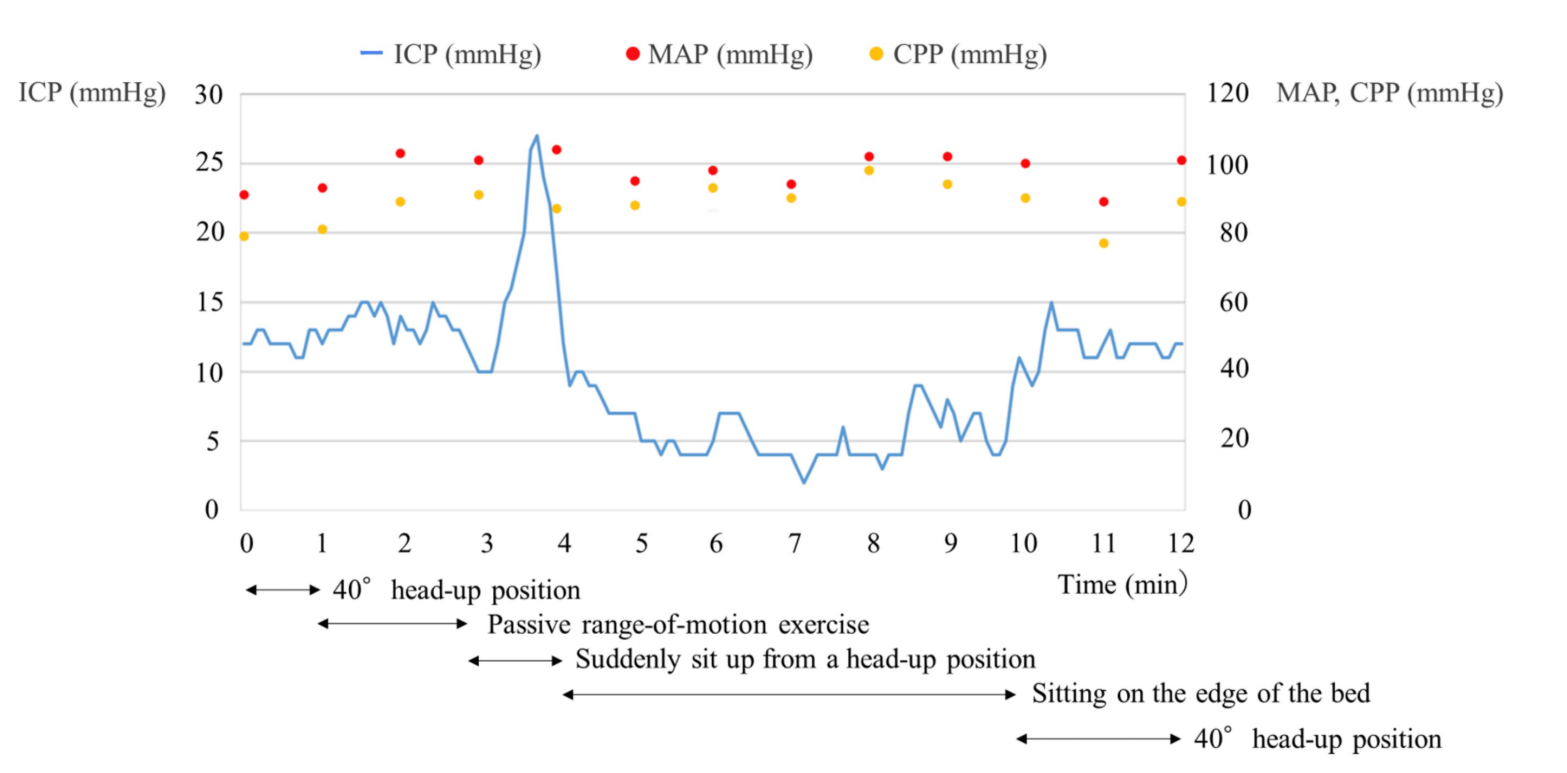
Changes in ICP, MAP and CPP during mobilization. ICP is presented as a blue solid line, MAP is presented as a red point, and CPP is presented as a yellow point. The positions and interventions at each time point are presented below the graph. ICP, intracranial pressure; MAP, mean arterial pressure; CPP, cerebral perfusion pressure.

A 47-year-old man collapsed at home and was brought to the hospital via an ambulance. He was diagnosed with a left putaminal hemorrhage and underwent emergency craniotomy for hematoma evacuation (day 1). The patient had a history of hypertension. Preoperative and postoperative brain computed tomography (CT) images are shown in Figure 1. After surgery, ICP monitoring was initiated owing to the risk of worsening cerebral edema, and ICP was measured using CODMAN ICP EXPRESS® (Integra Japan Inc., Tokyo, Japan). Oral amlodipine and intravenous nicardipine were initiated postoperatively for blood pressure control. No sedatives or anti-cerebral edema agents were administered. Physical therapy was initiated on day 2. The patient had a Glasgow Coma Scale score of E4V2M6 and was mildly somnolent; simple oral communication was possible. Motor paralysis of the right upper and lower limbs was severe; the patient was at the maximum assistance level for rolling over, sitting, and other activities. Mobilization (sitting on the edge of the bed) began on day 3 with the assistance of a physical therapist, with specific mobilization criteria set by a neurosurgeon. One of the key criteria for mobilization was ICP < 20 mmHg, with immediate cessation if sustained ICP elevation of > 20 mmHg occurred. ICP was sampled every 5 seconds. The mean arterial pressure (MAP) was continuously monitored via an arterial line, and the cerebral perfusion pressure (CPP) was calculated as CPP = MAP - ICP. The changes in ICP, MAP, and CPP during mobilization are shown in Figure 2. Before mobilization, ICP was well controlled at 13-15 mmHg, meeting the mobilization criteria. Joint range-of-motion exercises were performed at 40° head elevation. During the assisted transition to the sitting position, the patient suddenly attempted to rise independently, lifting the trunk off the bed. This caused the ICP to increase to 20-27 mmHg for approximately 20 seconds before returning to 13-15 mmHg. The ICP remained stable at approximately 5 mmHg while maintaining the sitting position for 6 minutes. The patient required constant assistance to maintain the sitting position. While the patient occasionally moved the non-paretic upper and lower limbs while sitting, there were no instances of coughing or noticeable body movements. After returning to the supine position, the ICP remained stable without further elevation. The MAP and CPP remained stable throughout the intervention period. ICP monitoring was discontinued on day 4, and the patient was transferred to another rehabilitation hospital on day 18.

## Discussion

ICP was evaluated over time during mobilization in a patient with putaminal hemorrhage under continuous ICP monitoring. While transitioning from the head-up position to the sitting position, the patient attempted to rise on his own, causing a transient increase in ICP above 20 mmHg. After sitting, the ICP decreased and remained at approximately 5 mmHg.

Few studies have investigated the effects of rehabilitation interventions on ICP. Previous studies have reported that ICP does not increase during interventions such as cycle ergometer use in the supine position [8] or passive range-of-motion exercises in the supine position [9]. Similarly, no sustained increase in ICP has been observed during mobilization in patients with intracerebral and subarachnoid hemorrhages [6,7]. However, these studies report only pre- and post-mobilization ICP and did not evaluate continuous ICP measurements during mobilization. In this case, there was no ICP elevation while sitting; instead, the ICP decreased and stabilized at approximately 5 mmHg. ICP is influenced by intracranial venous outflow, which tends to improve in the head-up position, thereby reducing ICP [10,11]. It is likely that transitioning to the sitting position facilitated a similar ICP-lowering effect in this case. However, because the MAP and CPP did not decrease, the decrease in ICP did not have any adverse effects.

One factor contributing to ICP elevation is the Valsalva effect [12]. The absence of limb exercises or active motion while the patient maintained the sitting position likely prevented ICP from increasing. However, when transitioning from the head-up position to the sitting position, the patient's effort to rise independently resulted in ICP surpassing 20 mmHg for approximately 20 seconds. During strenuous movements or activities with high physical exertion, the Valsalva effect can increase the ICP and warrants caution. However, ICP elevations above 20 mmHg sustained for > 5 minutes have been reported to be clinically significant [13]. Therefore, the transient ICP elevation observed in this case was unlikely to have had a significant impact. The intracerebral hemorrhage guidelines [14] do not specify a target CPP. However, traumatic brain injury guidelines recommend maintaining CPP at ≥ 60 mmHg [15]. In this case, CPP was maintained above 60 mmHg, suggesting that mobilization did not adversely affect CPP.

Several limitations of this case report should be considered when interpreting the results. First, mobilization was limited to the sitting position and was passive. The lack of active motion likely minimized the Valsalva effect and stabilized the ICP. Future studies should assess ICP changes during spontaneous movements such as standing or walking. Second, this report represents a single case with only one mobilization session. Further investigations involving large number of patients are required. Third, although brain edema was present postoperatively, the hematoma volume was small. In patients with intracerebral hemorrhage, differences in hematoma volume and their impact on ICP should be examined.

Although opportunities to monitor ICP during mobilization are rare, clinicians may encounter such cases in clinical practice. Determining the characteristics of ICP changes during mobilization in patients with putaminal hemorrhage may contribute to building evidence on the safety of early mobilization.

## Conclusions

Sitting on the edge of the bed was implemented in a patient with putaminal hemorrhage under ICP monitoring, and no sustained ICP elevation was observed while sitting. Further studies are needed to investigate various types and volumes of hematomas and to accumulate additional cases. If the trends in ICP fluctuations during mobilization are revealed, interventions that do not induce ICP fluctuations during mobilization may be possible, potentially contributing to improved safety.
